# Short-term outcomes associated with the use of a new powered circular stapler for rectal reconstructions: a retrospective study comparing it to manual circular staplers using inverse probability of treatment weight analysis

**DOI:** 10.1186/s12893-023-02218-w

**Published:** 2023-10-28

**Authors:** Nobuhisa Matsuhashi, Jesse Yu Tajima, Ryoma Yokoi, Shigeru Kiyama, Masahide Endo, Yuta Sato, Masashi Kuno, Hirokatsu Hayashi, Ryuichi Asai, Masahiro Fukada, Itaru Yasufuku, Yoshihiro Tanaka, Naoki Okumura, Katsutoshi Murase, Takuma Ishihara, Takao Takahashi

**Affiliations:** 1 Department of Gastroenterological Surgery and Pediatric Surgery, Gifu University Graduate School of Medicine, 1-1 Yanagido, Gifu City, 501-1194 Japan; 2 Innovative and Clinical Research Promotion Center, Gifu University Graduate School of Medicine, Gifu City, Japan

**Keywords:** Powered circular stapler, Rectal reconstructions, Short-term outcomes

## Abstract

**Background:**

The most common postoperative complication in malignant rectal surgery is anastomotic leakage (AL). AL after anterior or low anterior resection in rectal tumors is a fatal postoperative complication. Recently, the first automated suture circular stapler, which is expected to reduce the incidence of AL, (J&J).

**Materials and methods:**

This study included a total of 248 rectal tumor patients who underwent double stapler technique (DST) anastomotic procedures in the department of gastroenterological surgery /pediatric surgery at Gifu University School of Medicine from January 2017 to December 2021. The experience of a single institution utilizing the The Echelon circular™ stapler (ECP stapler:Manual VS Automatic) in rectal surgery cases was evaluated retrospectively from maintained database.

**Result:**

One hundred thirty-nine patients (58.4%) were performed by manual circular stapling, 99 patients (41.6%) by powerd circular stapling. Diverting stoma was performed in 45 cases (32.4%) by manual circular stapling, 42 patients (42.4%) by powerd circular stapling Postoperative complications were occurred clavien-dindo grade II or higher in 57 cases (23.9%) and grade III or higher in 20 cases (8.4%). Anastomotic leakage occurred in 14 patients (5.9%) within all grades. After IPTW, the variables of patient characteristics was SMD ≤ 0.2 (Table.3), and there was a significant difference in anastomotic leakage (Odds Ratio (OR), 0.57; 95% Confidence Interval(CI), 0.34–0.98 and there was a significant difference in anastomotic leakage (Odds Ratio (OR), 0.58; 95% Confidence Interval(CI), 0.34–0.98; *p* = 0.044). In addition, there was no significant difference in postoperative complications in grade II or higher (OR, 0.88; 95%CI, 0.65–1.18; *p* = 0.394) and grade III or higher (OR, 0.45; 95%CI, 0.28–0.73; *p* = 0.001) were significantly remarkable lower in powered circular stapling group.

**Conclusion:**

In this IPTW comparison of patients undergoing rectal reconstructions, the ECP trial cohort had lower risks of several surgical complications AL and statistically signifcant lower rates of ileus/bowel obstruction, infection, and bleeding as Clavien-Dindo ≥ grade II and III as compared with for whom manual circular staplers were used.

## Introduction

The most common postoperative complication in malignant rectal surgery is anastomotic leakage (AL). AL after anterior or low anterior resection in rectal tumors can be a fatal postoperative complication, and AL is said to increase the risk of local recurrence. The recent incidence of AL with stapler failure has been reported to range from 6.3% to 13.7%. Tissue tension may not be optimized, and manual circular staplers have been associated with technical errors and low satisfaction. Physicians with small hands or a weak grip, such as female physicians in particular, are less satisfied. However, the clinical results of this procedure have not yet been fully evaluated. We herein report the surgical short-term outcomes of circular stapler anastomosis performed at our institution.

In March of 2019, the first powered circular stapler expected to reduce the incidence of AL, the ECHELON CIRCULAR™ Powered Stapler (ECP; Ethicon Endo-Surgery, Inc., Cincinnati, OH, USA) was launched in the US and European markets. This powered circular stapler was introduced in Japan from 2018, and at present, we are actively using it in Gifu University Hospital.

A single-arm post-market multicenter trial to assess the intraoperative performance of the ECP during left-sided colectomy procedures was also recently completed [1]. In this trial, ECP showed few technical issues, a favorable safety profile, and ease of use for the creation of left-sided anastomoses as reported by operating surgeons. However, no comparisons were made with conventional manual circular staplers. Therefore, building upon the current state of evidence regarding the ECP, we conducted a retrospective comparison of clinical outcomes using a historical cohort of patients who underwent rectal resection using conventional manual circular staplers.

## Materials and methods

### Patients

#### Study outcome

This retrospective, single-center study included a total of 248 patients with rectal tumor who underwent double stapler technique anastomotic procedures in the Department of Gastroenterological Surgery/Pediatric Surgery at Gifu University School of Medicine from January 2017 to December 2021. The first subject consented on January, 2017, and the last subject’s final follow-up visit was in December, 2021.

The experience of a single institution utilizing the ECP stapler in manual vs. automatic mode in rectal surgery cases was evaluated retrospectively from a maintained database. The circular powered automatic stapler tested in this study was developed for easier, more stable firing and provides a 3D staple design to help create a more secure anastomosis. Preclinical analysis of this device showed reduced force required to fire, less movement during device application, and less leaking at the staple line compared to manual circular stapling. This study was designed assuming less leakage would occur with the ECP compared to manual staplers.

Eligible patients were aged 18–80 years with middle and low rectal adenocarcinoma and neuroendocrine tumor. Patients were assigned to receive robotic or conventional laparoscopic surgery with 139 patients undergoing manual stapling and 99 receiving automatic stapling. The staplers size were used 25 mm with 107 patients (automatic:65 patients:65.7%),29 mm with 120 patients (automatic:34 patients:34.3%)and other size with 11 patients (no automatic stapler)**.**All procedures included in the analysis used the ECP for creation of the stapled anastomoses.

### Surgical procedures

All cases were performed by laparoscopic or via robotic-assisted laparoscopic methods with Double stapler technique (DST) anastomosis. We used anastomosis instrument both the ECP stapler manual and automatic. All surgical procedures complied with the principles of total mesorectal excision or partial mesorectal excision. Lymph nodes at the origin of the inferior mesenteric artery were dissected. In the robotic group, the excision procedures and dissection of lymph nodes were performed by use of robotic techniques.

In all operations, the ECP was used to create the anastomosis according to the device’s instructions for use. Intra-operative details of the anastomotic reconstruction were recorded including the configuration. To operate the ECP stapler, the user presses a firing trigger that executes the powered firing sequence. A green light is illuminated to signal that firing is complete and the stapler can be removed. The evaluated ECP models were the 25-mm (CDH25P) and 29-mm (CDH29P) versions.

Leak tests with air insufflation into the rectum were completed to evaluate anastomotic integrity in every procedure. The decision to perform a diverting ileostomy was left to the discretion of the surgeon.

The study was completed after obtaining institutional review board approval (Approval no.:2019–197). The study protocol conformed to the ethical guidelines of the 1975 Declaration of Helsinki and the guidelines of the regional ethical committees of Zurich and Basel, Switzerland.

### Statistical analysis

Patient characteristics were too different to compare clinical outcomes in patients whose rectal tumors were resected laparoscopically or by robotic assistance without removing the confounding factors. Therefore, to adjust for baseline differences between the cohorts, the inverse probability of treatment weighting (IPTW) method was used. A multivariable logistic regression model based on age, sex, body mass index (BMI), ASA-PS (American Society of Anesthesiologists physical status), diabetes, prognostic nutritional index, operative period, operative blood loss, lateral pelvic lymph node dissection, tumor size, and cT factor and cN factor was used to estimate a propensity score for each patient. Missing values of all explanatory variables were imputed with a multiple imputation method generating 5 sets of imputed datasets based on aregImpute (www.rdocumentation.org/packages/Hmisc/versions/4.4-2/topics/aregImpute). The coefficients in the logistic regression model obtained from all 5 datasets were pooled by Rubin’s rules. Patient characteristics were compared before and after weighting by the inverse of the propensity score using, and the groups were considered homogeneous for each variable when the standardized mean difference (SMD) was ≤ 0.2. To assess whether there were differences in clinical outcomes between the two cohorts, we used a multivariable logistic regression model with weighting of each observation by IPTW. The variance of the coefficients was estimated using the Huber-White sandwich estimator to consider data clustering by the weighting. All statistical analyses were performed with a two-sided significance level of 5% and using R software version 4.2.2 (www.r-project.org).

## Results

### Patient characteristics

**Fig. 1:**
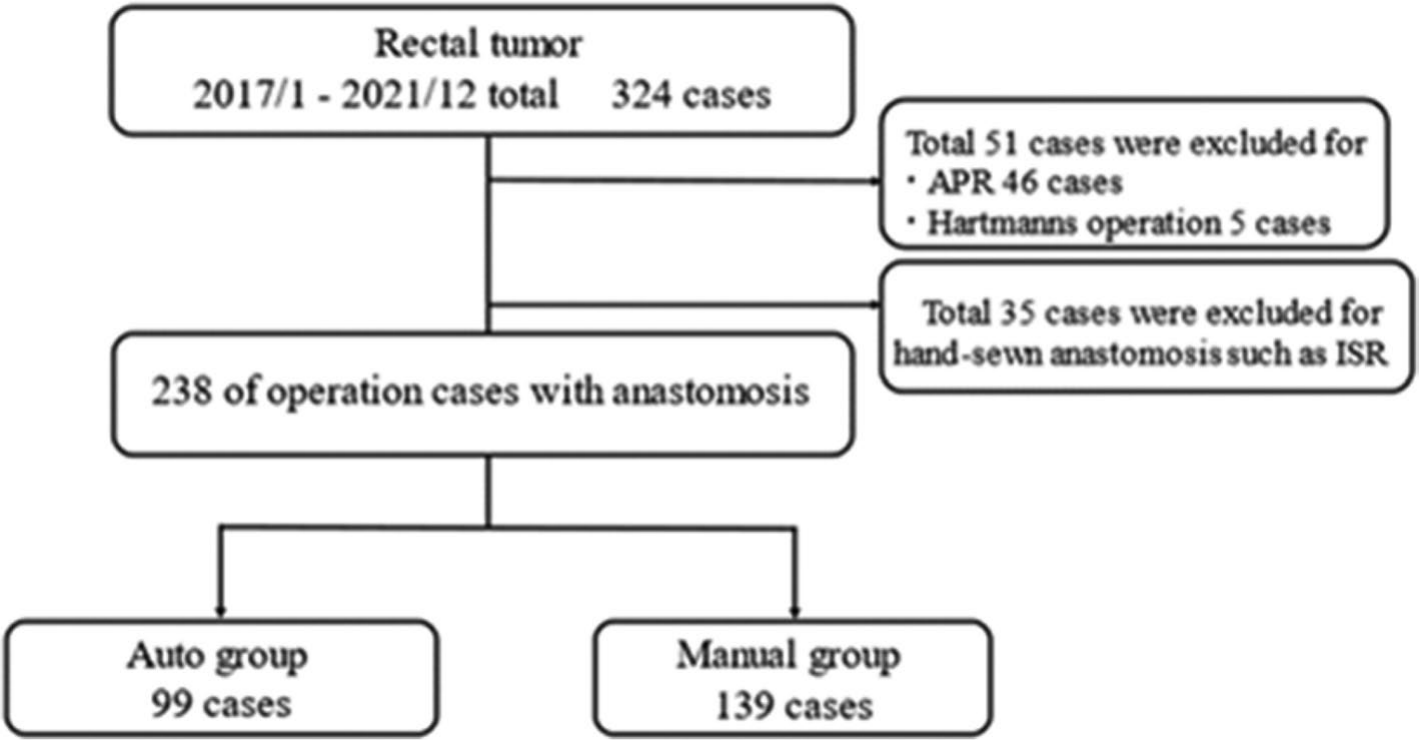
Flowchart of the study

Patient selection schemas are shown in Fig. 1. Between January 2017 and December 2021, 324 rectal tumors were resected laparoscopically or via robotic assistance. Of these, 238 cases in which anastomosis was performed were analyzed retrospectively. Total patient background characteristics andQuery oncological factors are shown in Table 1, and the perioperative factors are shown in Table 2. The median age of the patients was 67 years old, and 66.0% were male. Among the patients, 21 patients (8.8%)received preoperative treatment of neoadjuvant chemoradiotherapy and/or neoadjuvant chemotherapy. In details,15 patients (6.3%) were received preoperative treatment of neoadjuvant chemotherapy. 4 patients (1.6%) were received preoperative treatment of neoadjuvant chemoradiotherapy. 2 patients (0.8%) were received preoperative treatment of neoadjuvant chemotherapy plus chemoradiotherapy.In the anastomotic procedure, 139 patients (58.4%) underwent manual circular stapling, and 99 patients (41.6%) underwent powered circular stapling. A diverting stoma was performed in 45 cases (32.4%) by manual circular stapling and in 42 patients (42.4%) by powered circular stapling.

**Table 1:** Patient background and oncological factors

		**Auto (*N* = 99)**	**Manual (*N* = 139)**	***p*-value**†
Gender (Male, %)		66 (66.7)	91 (65.5)	0.96
Age (year)*		64.9 ± 11.0	65.5 ± 11.3	0.87
BMI (kg/m2)*		23.6 ± 3.55	23.0 ± 4.38	0.24
ASA-PS (%)	1	27 (27.3)	46 (33.1)	0.62
	2	67 (67.7)	88 (63.3)	
	3	5 (5.1)	5 (3.6)	
Diabetes (%)		22 (22.2)	23 (16.5)	0.35
PNI*		50.5 ± 5.5	51.6 ± 5.2	0.10
Histology (%)	Differentiated AC	92 (92.9)	125 (89.9)	0.71
	Undifferentiated AC	2 (2.0)	8 (5.8)	
	NET	4 (4.0)	3 (2.2)	
	Unknown	1 (1.0)	3 (2.2)	
tumor size (mm)*		36.6 ± 19.8	38.0 ± 19.9	0.54
cT factor (%)	1	24 (24.2)	31 (22.6)	0.98
	2	18 (18.2)	21 (15.1)	
	3	42 (42.4)	62 (44.6)	
	4	15 (15.2)	23 (16.5)	
cN factor (%)	0	54 (54.5)	85 (61.2)	0.30
	1	25 (25.3)	40 (28.8)	
	2	12 (12.1)	10 (7.2)	
	3	8 (8.1)	4 (2.9)	
cM factor (%)	0	86 (86.9)	129 (92.8)	0.19
	1	13 (13.1)	10 (7.2)	
Preoperative treatment (%)	NAC	13 (5.5)	2 (0.8)	0.002
	CRT	2 (0.8)	2 (0.8)	
	NAC + CRT	1 (0.4)	1 (0.4)	

*BMI* Body Mass Index

*ASA-PS* American Society of Anesthesiologists physical status

*PNI* Prognostic Nutritional Index = (10 × Alb) + (0.005 × TLC)

*AC* Adenocarcinoma

*NET* Neuroendocrine tumor

*NAC* Neoadjuvant Chemotherapy

*CRT* Chemoradiotherapy

*AL *Anastomotic Leakage

*Mean ± SD, †Pearson's Chi-squared test

**Table 2:** Demographic and surgical characteristics of the patient

		**Auto (*N* = 99)**	**Manual (*N* = 139)**	***p*-value**†
Operative period (min)*		265.7 ± 97.8	290.9 ± 109.2	0.063
Operative blood loss (ml)*		24.8 ± 56.1	32.9 ± 82.9	0.37
Procedure (%) Robot		67 (67.7)	27 (19.4)	< 0.001
Lap		32 (32.3)	112 (80.6)	
Size of Stapler 25 mm		65 (65.7)	42 (30.2)	< 0.001
29 mm		34 (34.3)	86 (61.9)	
other		0 (0)	11 (7.9)	
LPLN dissection (%)		6 (6.1)	9 (6.5)	1.0
Number of lymph nodes collection *		18.7 ± 9.4	18.7 ± 10.2	0.83
Diverting stoma (%)	Total	42 (42.4)	45 (32.4)	0.15
	Ileostomy	13 (13.1)	26 (18.7)	0.022
	Colostomy	29 (29.3)	19 (13.7)	
First defecation (day)*		4.2 ± 2.9	3.9 ± 2.3	0.12
Postoperative complication (%)**	All grade	34 (34.3)	43 (30.9)	0.68
	≥ Grade II	23 (23.2)	34 (24.5)	0.83
	≥ Grade III	5 (5.1)	15 (10.8)	0.12
	AL	3 (3.0)	11 (7.9)	0.12
Postoperative hospital stay (day)*		14.4 ± 6.0	14.4 ± 6.8	0.79

*LPLN* Lateral Pelvic Lymph Node

*Mean±SD, †Pearson’s Chi-squared test

**Clavien-Dindo grade

### Association between surgical approach and short-term outcome

There were no significant differences in intraoperative blood loss, tumor size, preoperative T stage, N stage, and the number of lymph nodes dissected between the powered circular stapling group and the manual circular stapling group. Postoperative complications of Clavien-Dindo grade II or higher occurred in 57 cases (23.9%) and those of grade III or higher occurred in 20 cases (8.4%). Anastomotic leakage occurred in 14 patients (5.9%) within all complication grades (Table 2).

After IPTW, the variables of the patient characteristics with SMD ≤ 0.2 were revealed (Table 3), which showed a significant difference in AL between the two stapling groups (odds ratio [OR], 0.58; 95% confidence interval [CI], 0.34–0.98; *p* = 0.044). However, there was no significant difference in postoperative complications of grade II or higher (OR, 0.88; 95% CI, 0.65–1.18; *p* = 0.394), and complications of grade III or higher (OR, 0.45; 95% CI, 0.28–0.73; *p* = 0.001) were significantly and remarkably lower in the powered circular stapling group (Table 4).

**Table 3:**
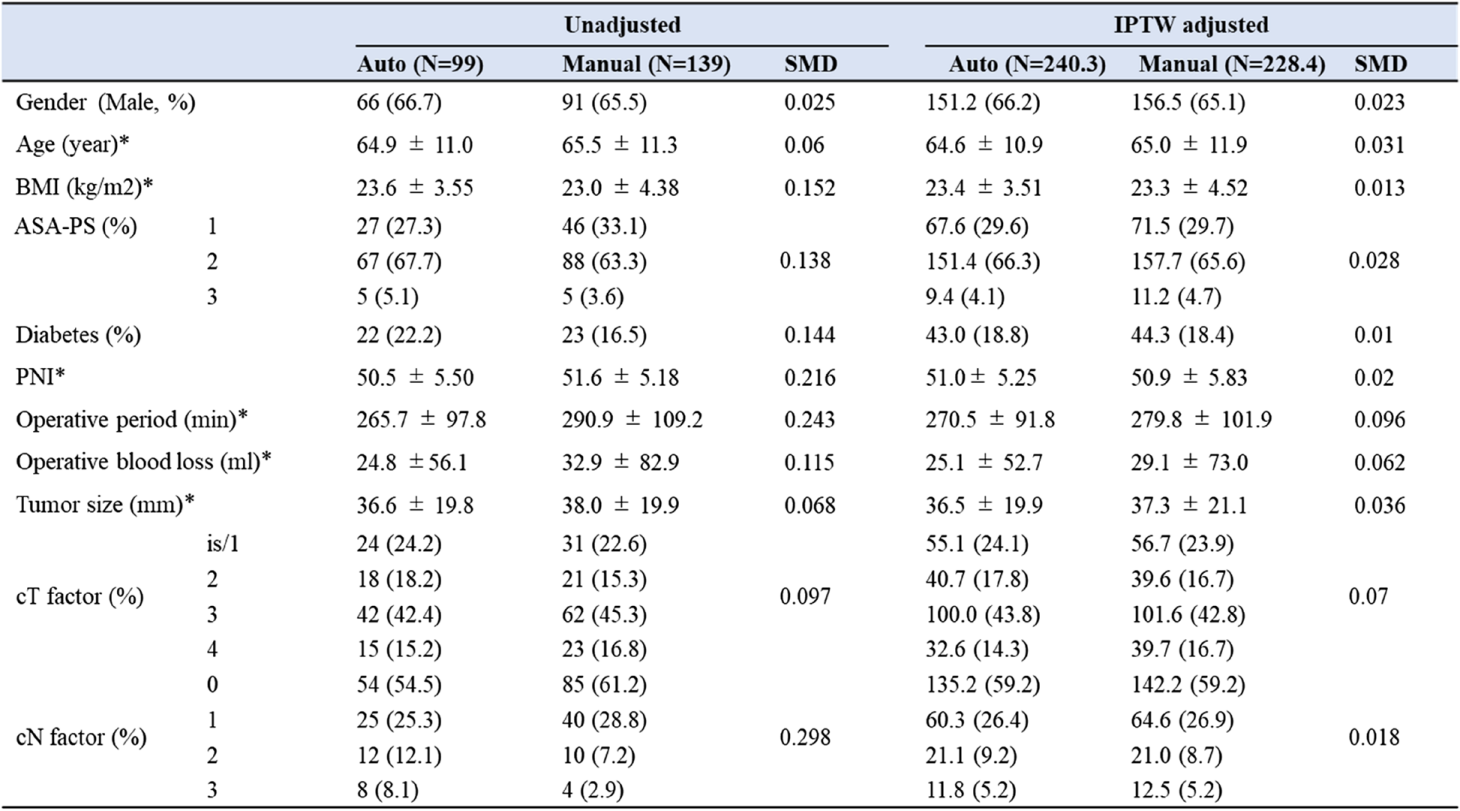
IPTW propensity score matching

*BMI* Body Mass Index

*ASA-PS* American Society of Anesthesiologists physical status

*PNI* Prognostic Nutritional Index = (10 × Alb) + (0.005 × TLC)

*Mean ± SD, †Pearson’s Chi-squared test

**Table 4:** Risk of postoperative complications in Auto group against Manual group

Postoperative complication		Odds Ratio	95%CI	*p*-value †
Clavien-Dindo grade	≥ grade II	0.88	0.65–1.18	0.394
	≥ grade III	0.45	0.28–0.73	0.001
Anastomotic leakage	(All grade)	0.58	0.34–0.98	0.044

after the IPTW propensity score matching

^†^Pearson's Chi-squared test

## Discussion

Laparoscopic surgery has been shown to be associated with higher complication rates when performed for rectal cancer than when performed for colon cancer because of technical difficulties and anatomical limitations in the pelvic cavity. According to the results of the CLASICC trial, the complication rate for rectal surgery was 13%, nearly twice higher than the 7% rate for colon surgery. In addition, the rate of AL was higher for rectal surgery (10.2%) than for colon surgery (2.3%; *p* < 0.001) [2]. Furthermore, postoperative morbidities can delay the administration of adjuvant therapy, increase the hospital stay, and reduce cost-effectiveness [3–5].

Other publications reporting lower rates usually originated from dedicated centers. Analyses adjusted for confounding factors revealed male sex and high ASA grade as independent risk factors for AL, which is consistent with the literature. Other reported predictors of AL, such as high BMI, were not confirmed in the present analysis. Another well known risk factor for AL is comorbidity, reflected in the Charlson score and ASA classification in the present study [6].

In surgical procedures, it is important to note that ???? has been reported to increase the number of linear staples used for rectal transection and was a risk factor for AL [7]. Some previous studies reported that the number of linear staples used for rectal transection was also a risk factor for AL [1]. There is concern that an increased number of staple firings may lead to small defects between staple lines and cause AL. Furthermore Kim et al. found that more than two staple firings was associated with AL [8], and Fukada et al. reported that the number of linear staples used was significantly higher in males, patients with a tumor closer to the rectal verge, and longer operation times [9]. At our institution, we have also reported an increase in AL when the number of linear staple firings exceeds three times. The most important thing the surgeon can do is to reduce the number of staplers.

In surgical procedures, some reports consider tension and the blood flow to the anastomosis to be even more important. To relieve tension at the anastomosis, mobilization of the colon of the proximal side is important. Sometimes, additional maneuver of the splenic flexure may be performed, but it is important to dissect on the proximal side of the colon and the distal anal rectal side to avoid tension on the anastomosis [2].

This study assessed outcomes of the ECP trial cohort as indirectly compared with a retrospectively established historical cohort of patients undergoing rectal reconstructions with manual circular staplers [10–12].

Recently, indocyanine green fluorescence angiography (ICG FA) technology has gradually been applied to colorectal surgery and is showing promising results in reducing the incidence of AL. ICG is a near-infrared fluorescent dye that can be detected by imaging systems [13]. The mechanism of ICG FA to prevent AL is to reveal areas in the anastomosis with insufficient blood supply. If vascular perfusion via ICG FA is poor or delayed, the transection line of the proximal bowel must be shifted to a site with good vascular perfusion, and the anastomosis is performed at the changed transection line. A few studies have applied this novel method to prevent AL in colorectal surgery and have shown promising results [14, 15]. In our institution, we also perform oral-side colon transfer to avoid tension at the anastomosis. Furthermore, we confirm that there is no tension at the time of the anastomosis and also check the blood flow in the oral side colon using ICG before anastomosis.

From this IPTW-adjusted comparison framework, use of the ECP was associated with significantly lower rates (incidence proportions) of several postoperative complications as well as of 30-day readmissions.

In rectal procedures, the rate of AL in this study was lower for the powered circular stapler than for the manual circular stapler. For all types of adverse events observed in our analysis, including serious adverse events.

Postoperative complications of Clavien-Dindo grade II or higher occurred in 23.9% and those of grade III or higher occurred in 8.4% of patients. Across all Clavien-Dindo grades, AL occurred in 5.9% of the patients.

There was significant difference in AL (OR 0.58, 95% CI 0.34–0.98, *p* = 0.044). In addition, there was no significant difference in postoperative complications of grade II or higher (OR 0.88, 95% CI 0.65–1.18, *p* = 0.394), and those of grade III or higher (OR 0.45, 95% CI 0.28–0.73, *p* = 0.001) were significantly and remarkably lower in the powered circular stapling group.

Daniel et al. reported that mean grip strength was significantly greater for male surgeons, although ease of use of the ECP was judged to be nearly equally high by surgeons of both genders, indicating that grip strength is not a factor for effective use of the device. It was designed specifically for stability during firing and generation of consistent compression, which may contribute to fewer staple-line leaks [16].

In our study, the primary surgeon was a qualified surgeon certified by the Endoscopic Surgical Skill Qualification System of the Japanese Society of Endoscopic Surgery or an instructor was always present as the first assistant surgeon. Therefore, there was no difference in this study in regard to technical ability of the surgeons. Also, in terms of historical background, the study was limited to a recent 5-year period as this has been a period of relatively standardized technology with no changes in devices used in laparoscopic surgery. We thought it would be meaningful to examine AL by manual and ECP stapling during this period and that it would be possible to clarify whether manual circular staplers or ECP are better for AL in a retrospective study.

Pollack E reported that The ECP was also associated with 27 fewer length of stay (LOS) days, 0.38 fewer readmissions and 0.22 fewer non-home discharges related to anastomotic leaks annually. The incremental cost burden to a hospital that upgrades to ECP for 100 cases a year is estimated to be $11,400—a cost that is easily offset by preventing 1–2 anastomotic leak complications, given the mean incremental cost estimates for this complication [17].

In addition, we decided to conduct an accurate statistical study using an IPTW comparison between these two groups.

The primary limitation of this report is the lack of a randomized comparison between the ECP stapler and a control circular stapler. The second limitation of this report is the problem that it is combined to performed by laparoscopic or via robotic-assisted laparoscopic methods. But it was no significant difference in anastomotic leakage between robotic-assisted and laparoscopic surgery in our study (data not shown).

However, our objective was to obtain an accurate estimation of technical issues and complications and adverse events associated with use of the novel ECP device.

## Conclusion

In this IPTW comparison of patients undergoing rectal reconstructions, the ECP trial cohort had lower risks of several surgical complications of AL and statistically significant lower rates of ileus/bowel obstruction, infection, and bleeding as complications of Clavien-Dindo ≥ grades II and III as compared with the patients in whom manual circular staplers were used. Further controlled prospective clinical studies are needed to confirm the validity of this finding.

## Data Availability

All data is provided in the manuscript.

## Abbreviations

AL: Anastomotic leakage
BMI: Body mass index
CI: Confidence interval
ECP: ECHELON CIRCULAR™ Powered Stapler
ICG FA: Indocyanine green fluorescence angiography
IPTW: Inverse probability of treatment weighting
OR: Odds ratio
SMD: Standardized mean difference
