# Correction: Short-term outcomes associated with the use of a new powered circular stapler for rectal reconstructions: a retrospective study comparing it to manual circular staplers using inverse probability of treatment weight analysis

**DOI:** 10.1186/s12893-023-02278-y

**Published:** 2023-12-19

**Authors:** Nobuhisa Matsuhashi, Jesse Yu Tajima, Ryoma Yokoi, Shigeru Kiyama, Masahide Endo, Yuta Sato, Masashi Kuno, Hirokatsu Hayashi, Ryuichi Asai, Masahiro Fukada, Itaru Yasufuku, Yoshihiro Tanaka, Naoki Okumura, Katsutoshi Murase, Takuma Ishihara, Takao Takahashi

**Affiliations:** 1 Department of Gastroenterological Surgery and Pediatric Surgery, Gifu University Graduate School of Medicine, 1-1 Yanagido, Gifu City, 501-1194 Japan; 2 Innovative and Clinical Research Promotion Center, Gifu University Graduate School of Medicine, Gifu City, Japan

**Correction: BMC Surg 23; 1 (2023)**

**https://doi.org/10.1186/s12893-023-02218-w**

Following publication of the original article [1], there are some text change in the abstract section under Result the paragraph should need to change from “Result One hundred thirty-nine patients (58.4%) were performed by manual circular stapling, 99 patients (41.6%) by powerd circular stapling. Diverting stoma was performed in 45 cases (32.4%) by manual circular stapling, 42 patients (42.4%) by powerd circular stapling Postoperative complications were occurred clavien-dindo grade II or higher in 57 cases (23.9%) and grade III or higher in 20 cases (8.4%). Anastomotic leakage occurred in 14 patients (5.9%) within all grades. After IPTW, the variables of patient characteristics was SMD ≤ 0.2 (Table 3), and there was a significant difference in anastomotic leakage (Odds Ratio (OR), 0.57; 95% Confidence Interval(CI), 0.34–0.98; *p* = 0.041). In addition, there was no significant difference in postoperative complications in grade II or higher (OR, 0.88; 95%CI,0.65–1.19; *p* = 0.417) and grade III or higher (OR, 0.46; 95%CI, 0.29–0.74; *p* = 0.001) were significantly remarkable lower in powered circular stapling group.” to “ One hundred thirty-nine patients (58.4%) were performed by manual circular stapling, 99 patients (41.6%) by powerd circular stapling. Diverting stoma was performed in 45 cases (32.4%) by manual circular stapling, 42 patients (42.4%) by powerd circular stapling Postoperative complications were occurred clavien-dindo grade II or higher in 57 cases (23.9%) and grade III or higher in 20 cases (8.4%). Anastomotic leakage occurred in 14 patients(5.9%) within all grades. After IPTW, the variables of patient characteristics was SMD ≤ 0.2 (Table 3),and there was a significant difference in anastomotic leakage (Odds Ratio (OR), 0.58; 95% Confidence Interval(CI), 0.34–0.98; *p* = 0.044). In addition, there was no significant difference in postoperative complications in grade II or higher (OR, 0.88; 95%CI, 0.65–1.18; *p* = 0.394) and grade III or higher (OR, 0.45; 95%CI, 0.28–0.73; *p* = 0.001) were significantly remarkable lower in powered circular stapling group.”

In addition, there is a change in the Fig. 1 and the same has shown below:

**Fig. 1:**
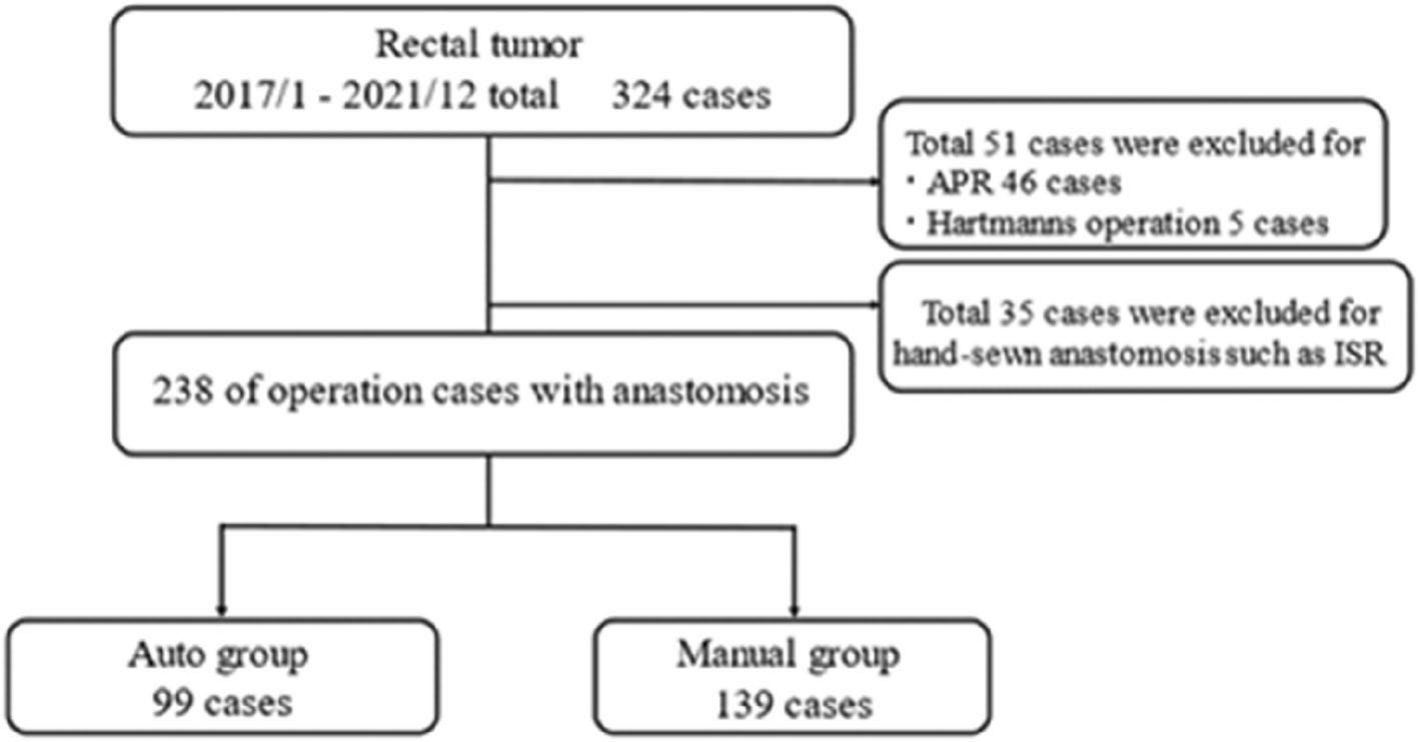
Flowchart of the study

The original article has been corrected.
